# Central serotonin-2A (5-HT2A) receptor dysfunction in depression and epilepsy: the missing link?

**DOI:** 10.3389/fphar.2015.00046

**Published:** 2015-03-17

**Authors:** Bruno P. Guiard, Giuseppe Di Giovanni

**Affiliations:** ^1^CNRS, Centre de Recherches sur la Cognition Animale, UMR 5169, ToulouseFrance; ^2^CNRS, Centre de Recherches sur la Cognition AnimaleUniversité de Toulouse 3, UMR 5169, Toulouse, France; ^3^INSERM U1178 Team ≪Depression and Antidepressants≫Faculté de Pharmacie Paris Sud, Châtenay-Malabry, France; ^4^Neurophysiology Unit, Laboratory for the Study of Neurological Disorders, Department of Physiology and Biochemistry, University of Malta, MsidaMalta; ^5^School of Biosciences, University of Cardiff, CardiffUK

**Keywords:** 5-HT, 5-HT_2A_ receptor, antidepressants, antipsychotics, depression, epilepsy

## Abstract

5-Hydroxytryptamine 2A receptors (5-HT_2A_-Rs) are G-protein coupled receptors. In agreement with their location in the brain, they have been implicated not only in various central physiological functions including memory, sleep, nociception, eating and reward behaviors, but also in many neuropsychiatric disorders. Interestingly, a bidirectional link between depression and epilepsy is suspected since patients with depression and especially suicide attempters have an increased seizure risk, while a significant percentage of epileptic patients suffer from depression. Such epidemiological data led us to hypothesize that both pathologies may share common anatomical and neurobiological alteration of the 5-HT_2A_ signaling. After a brief presentation of the pharmacological properties of the 5-HT_2A_-Rs, this review illustrates how these receptors may directly or indirectly control neuronal excitability in most networks involved in depression and epilepsy through interactions with the monoaminergic, GABAergic and glutamatergic neurotransmissions. It also synthetizes the preclinical and clinical evidence demonstrating the role of these receptors in antidepressant and antiepileptic responses.

## The 5-HT_2A_-Rs: Distribution in Brain Areas Releted to Depression and Epilepsy and their Pharmacological Properties

**FIGURE 1:**
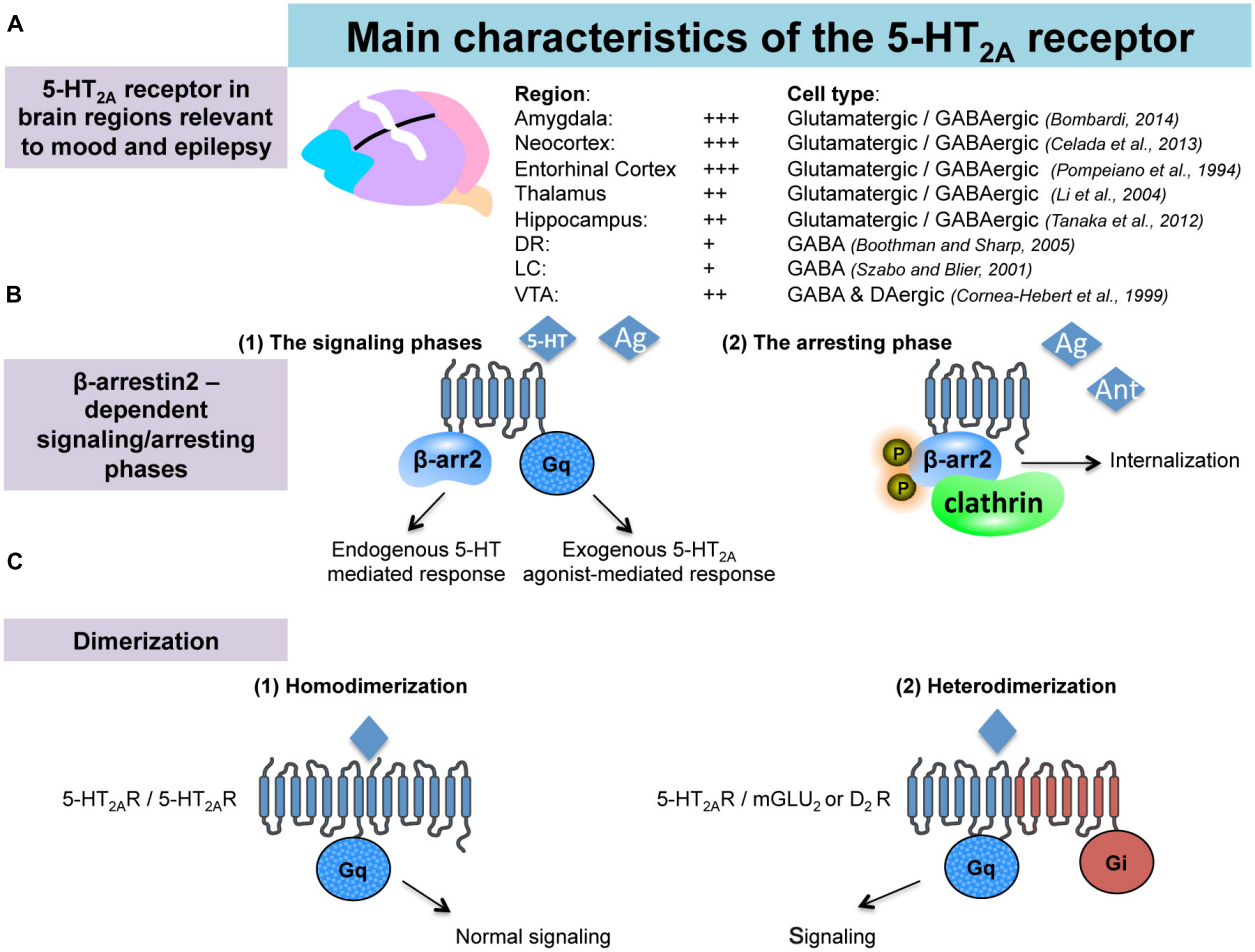
**Anatomical and pharmacological properties of the 5-HT2A receptors in the brain. (A)** 5-HT_2A_ receptors (5-HT_2A_-Rs) are located in brain regions involved in emotionality and epilepsy. **(B)** Interactions between 5-HT_2A_-Rs and beta-arrestin 2. According to the nature of the 5-HT_2A_-Rs agonist (endogenous/exogenous), 5-HT_2A_-Rs-mediated signaling may recruit beta-arrestin2-dependant or -independent pathways (signaling phases). Such a beta-arrestin2 is also involved in the down regulation/internalization of the 5-HT_2A_-Rs (arresting phase). **(C)** Dimerization of the 5-HT_2A_-Rs with GPCRs is necessary to activate signaling pathway.

Serotonin is an important modulator of a plethora of physiological functions in the brain. The diverse 5-HT effects are mediated by seven classes of 5-HT receptors (5-HT-Rs) and, at least, 15 subtypes (Barnes and Sharp, 1999). Pharmacological and genetic studies have highlighted an important role for 5-HT_2A_-Rs in specific CNS pathologies including depression and epilepsy. 5-HT_2A_-Rs are members of the metabotropic seven transmembrane-spanning receptors superfamily frequently referred to as GPCRs. In particular, 5-HT_2A_-Rs belong to the 5-HT_2_ subfamily consisting, with 5-HT_2B_ and 5-HT_2C_, of three Gq/G11-coupled receptors, which mediate excitatory neurotransmission (Millan et al., 2008). Using *in situ* hybridization, western blot and immunohistochemical analyses in rodents, 5-HT_2A_-R mRNA or the protein have been identified in various brain regions involved in emotionality and epilepsy, such as the amygdala, the hippocampus (Bombardi, 2012; Tanaka et al., 2012), the thalamus (Li et al., 2004) as well as in several cortical areas (entorhinal, cingulate, piriform, and frontal cortices Pompeiano et al., 1994; Santana et al., 2004; Amargos-Bosch et al., 2005; de Almeida and Mengod, 2007; **Figure 1A**). 5-HT_2A_-Rs have also been detected in all monoaminergic brainstem levels; i.e., the MRN/DRN, the LC and the VTA (Cornea-Hebert et al., 1999; Doherty and Pickel, 2000; Nocjar et al., 2002; Quesseveur et al., 2012; **Figure 1A**), which also strongly suggests their indirect role in mood and depression by regulating the monoaminergic systems. Indeed, 5-HT_2A_-Rs act at the monoaminergic somatodendritic or nerve terminals levels either through a direct or indirect action involving glutamatergic and/or GABAergic neurons (Di Giovanni, 2013).

A major feature of the 5-HT_2A_-Rs lies in their interactions with β-arrestin. Previous work showed that the 5-HT_2A_-Rs colocalize with β-arrestin-1 and -2 in cortical neurons (Gelber et al., 1999). Interestingly, it has been shown in β-arrestin-2 KO mice (β-Arr2^-/-^), in which 5-HT_2A_-Rs were predominantly localized to the cell surface, that 5-HT was no longer capable of inducing behavioral responses (i.e., head-twitch). These observations suggested that β-arrestin-2 mediates intracellular trafficking of the 5-HT_2A_-Rs (**Figure 1B**), and that the cellular events play a role in the induction of head-twitch in response to elevated 5-HT levels. Alternatively, the authors found that the preferential 5-HT_2A_-R agonist DOI still produces the head-twitch in β-Arr2^-/-^ mice thereby suggesting that β-arrestins are not required for DOI-mediated response (Abbas and Roth, 2008; Schmid et al., 2008). These data emphasize the contribution of the nature of the ligand in determining the receptor signaling pathway and, ultimately, the physiological responses induced by the compound. 5-HT_2A_Rs coupling to the intracellular scaffolding proteins β-arrestins can either dampen or facilitate GPCRs signaling, and therefore, represent a key point at which receptor signaling may diverge in response to particular ligands (**Figure 1B**).

There is another mechanism by which the 5-HT_2_-Rs subtypes can regulate their signaling. Recent evidence demonstrates that these receptors can form stable homo- (Herrick-Davis et al., 2005; Brea et al., 2009) and heteromeric complexes with other types of GPCRs including the mGluR2 and D_2_-DA Rs (González-Maeso et al., 2008; Albizu et al., 2011; Fribourg et al., 2011; Lukasiewicz et al., 2011; Moreno et al., 2011; Delille et al., 2012; Moreno et al., 2012; **Figure 1C**). The *in vivo* functional consequences of such oligo-dimerization of 5-HT_2A_-Rs has yet to be determined but this process is likely responsible for changes in binding and coupling properties of the receptors. Supporting this hypothesis, it has been reported that head-twitch induced by the preferential 5-HT_2A_-R agonists lysergic acid diethylamide (LSD) and DOI is completely abolished in mGlu2 knock-out (mGlu2^-/-^ KO) mice (González-Maeso et al., 2007; Moreno et al., 2011, 2012; González-Maeso, 2014).

Both examples illustrate the fact that the functional activity of the 5-HT_2A_-Rs is finely regulated, notably through its interactions with β-arrestin-2 or other GCPRs at the cell membrane. A better knowledge of the physiological relevance of such interactions may help identify new strategies to modulate 5-HT_2A_-Rs-mediated transmission.

## The 5-HT_2A_-Rs in the Modulation of Neurotransmission

### GABA/Glutamate

Serotonergic neurotransmission and more particularly activation of post-synaptic 5-HT_2A_-Rs in the PFC play a pivotal role in the regulation of the neuronal activity of this brain region. As mentioned in the first part of this review, a substantial proportion of excitatory pyramidal neurons express the 5-HT_2A_-R mRNA (Santana et al., 2004; Amargos-Bosch et al., 2005; de Almeida and Mengod, 2007), while these mRNAs are also present in ~25% of GAD-containing cells (Santana et al., 2004). Functional *in vitro* studies showed that 5-HT increased glutamatergic spontaneous excitatory post-synaptic currents (EPSCs) in pyramidal neurons in layer V of the PFC and this effect was mediated by 5-HT_2A_-Rs (Aghajanian and Marek, 1999; Celada et al., 2013). Interestingly, intracellular recordings from pyramidal neurons in layers V and VI of the rat mPFC indicated that the application of the 5-HT_2A/2C_-R agonist DOB produced a biphasic modulation of *N*-methyl-D-aspartate (NMDA)-induced responses, e.g., membrane depolarization, bursts of action potentials and inward current (Arvanov et al., 1999). Indeed, DOB facilitated and inhibited NMDA responses at low and higher concentrations, respectively while these effects were blocked by the 5-HT_2A_-R antagonist MDL100907 (Aghajanian and Marek, 1997; Arvanov et al., 1999). These results confirmed a previous report showing that iontophoretic application of DOI at low and high ejecting currents facilitated and inhibited, respectively, glutamate-evoked firing rates of pyramidal cells in the mPFC (Ashby et al., 1990) thereby demonstrating the complex regulation of these cells by 5-HT_2A_-Rs. *In vivo*, the systemic administration of DOI has been shown to affect the firing rate of pyramidal neurons, since it produced both cell excitation and inhibition (Puig et al., 2003). It is possible that the inhibition of pyramidal neurons by DOI concerns a sub-population of cells innervated by 5-HT_2A_-Rs-expressing GABAergic interneurons. Consistent with this hypothesis, the intra-cortical injection of DOI dose-dependently increased local extracellular GABA levels in rats while systemic DOI administration resulted in Fos protein expression in GAD67-immunoreactive interneurons of the PFC (Abi-Saab et al., 1999). It has also been demonstrated that the local application of DOI in the mPFC increased 5-HT release (Martin-Ruiz et al., 2001; Bortolozzi et al., 2003; Amargos-Bosch et al., 2004). Such elevation in cortical 5-HT outflow produced local glutamate release (Mocci et al., 2014) and subsequent activation of α-amino-3-hydroxy-5-methyl-4-isoxazolepropionic acid (AMPA)/NMDA receptor located on the 5-HT nerve terminals. In agreement with this hypothesis the DOI-induced increase in cortical 5-HT outflow was reversed by NBQX (an AMPA-KA antagonist) but not by MK-801 (a NMDA antagonist; Martin-Ruiz et al., 2001). Altogether, these findings indicate that 5-HT and glutamate positively interact in the PFC and both have a tendency to become self-reinforcing.

If the activation of the 5-HT_2A_-Rs mainly stimulates the activity of excitatory pyramidal neurons, an interaction with inhibitory GABAergic neurons is also possible not only in the PFC but also in other serotonergic nerve terminal regions regulating mood-related behavior.

rs. For example, in the PAG area, the stimulation of 5-HT_2A_-Rs was shown to cause a panicolytic-like effect that is mediated by facilitation of GABAergic neurotransmission (de Oliveira Sergio et al., 2011). In the amygdala, double immunofluorescence labeling demonstrated that the 5-HT_2A_-Rs are primarily localized to parvalbumin-containing interneurons suggesting that 5-HT primarily acts via 5-HT_2A_-R to facilitate BLA GABAergic inhibition (Jiang et al., 2009). Accordingly, alpha-methyl-5-HT, a 5-HT_2_-Rs agonist, enhanced frequency and amplitude of spontaneous inhibitory post-synaptic currents (sIPSCs) recorded on the BLA neurons *in vitro*, and this effect was blocked by selective 5-HT_2A_-R antagonists (Jiang et al., 2009). In the hippocampus, the activation of 5-HT_2A_-Rs has also been proposed to increase GABAergic synaptic activity in the CA1 region (Shen and Andrade, 1998).

### Monoamines

**FIGURE 2:**
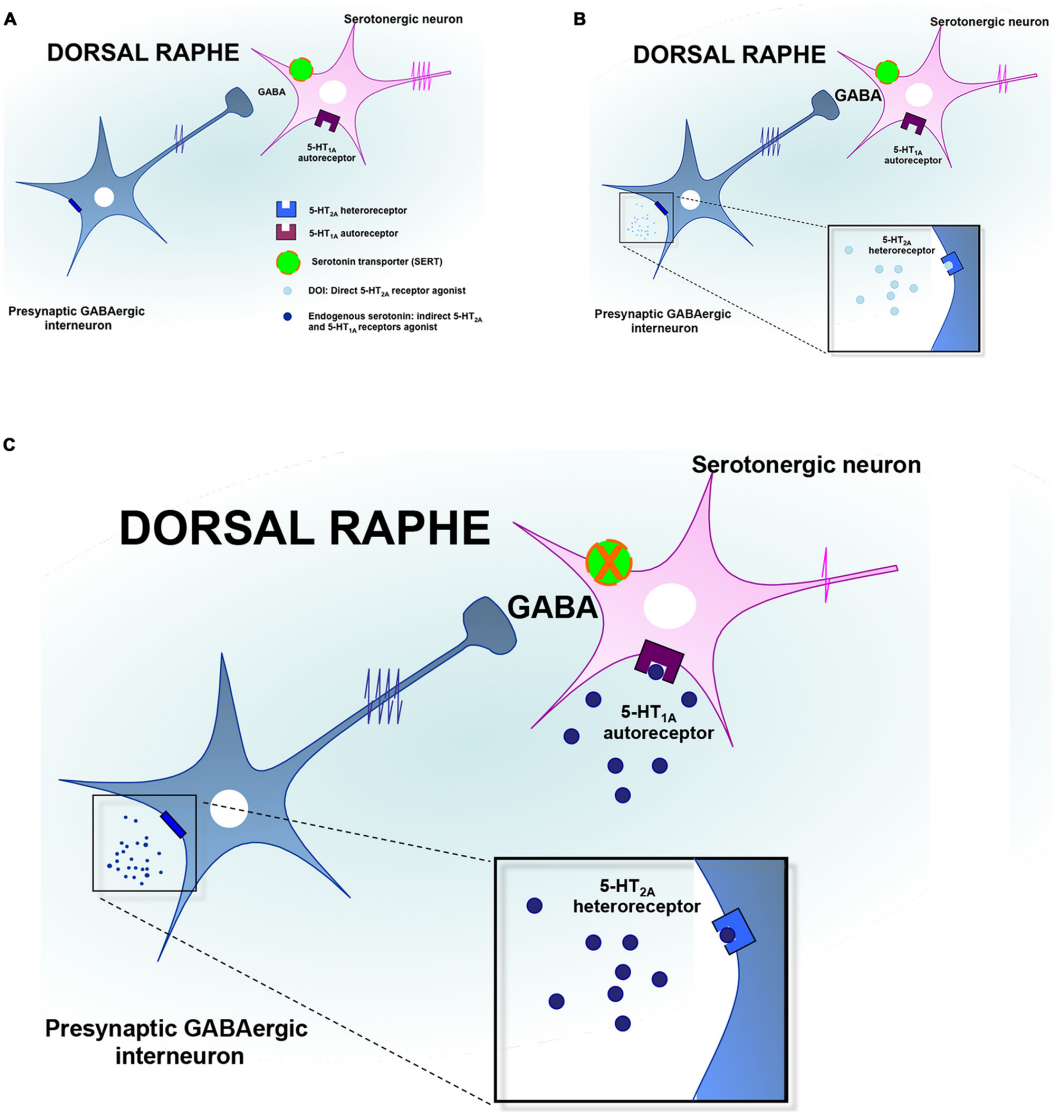
**Regulation of serotonin neuronal activity by the 5-HT_2A_ receptors in the dorsal raphe. (A)** The dorsal raphe nucleus (DRN) contains GABAergic interneurons expressing the 5-HT_2A_ receptors (5-HT_2A_-Rs) and which project on serotonergic neurons cell bodies to regulate their firing activity. **(B)** In response to the activation of 5-HT_2A_-Rs by preferential agonists such as DOI, the neuronal activity of GABAergic interneurons is increased leading to an accumulation of GABA in the synaptic cleft. Such an elevation of GABAergic tone contributes to inhibit DRN 5-HT neurons discharge. **(C)** In response to the administration of SSRIs, endogenous serotonin activates the 5-HT_1A_ autoreceptors and the 5-HT_2A_ heteroreceptors thereby producing additional inhibitory influences onto DRN 5-HT neurons to silence their activity.

As mentioned earlier, immunoreactivity for the 5-HT_2A_-Rs has been identified in the DRN and more particularly on GABAergic interneurons (Xie et al., 2002; Serrats et al., 2005). It should be noted that serotonergic raphe nuclei receive a prominent GABAergic input via distant sources as well as interneurons (Harandi et al., 1987; Bagdy et al., 2000; Gervasoni et al., 2000; Varga et al., 2001; Vinkers et al., 2010), and functional evidence suggests that the activation of GABA release in the DRN may be under the control of the 5-HT_2A_-Rs (**Figure 2**). Indeed, it has been reported that the activation of these receptors increased Fos expression in GAD-positive DRN neurons (Boothman and Sharp, 2005; Quérée et al., 2009). Accordingly, *in vitro* studies demonstrated that the local application of DOI in this brain region induces a dose-dependent increase in the frequency of inhibitory post-synaptic currents (IPSCs; Liu et al., 2000; Gocho et al., 2013). *In vivo* recordings in the DRN showed that the systemic administration of DOI attenuated the firing rate of 5-HT neurons (Wright et al., 1990; Garratt et al., 1991; Martin-Ruiz et al., 2001; Boothman et al., 2003; Bortolozzi et al., 2003; Boothman and Sharp, 2005; Quesseveur et al., 2013). In a recent study, we extended these observations to the fact that the 5-HT_2A_-Rs also played an important role in the acute electrophysiological response to SSRIs. Indeed, since it has long been recognized that the inhibitory effect of SSRI on 5-HT firing rate was mediated by the overactivation of somatodendritic 5-HT_1A_ autoreceptor in the DRN (Gardier et al., 1996), we blocked this mechanism by using the 5-HT_1A_-R antagonist WAY100635 (Quesseveur et al., 2013). In these conditions, the inhibitory effects of SSRI escitalopram on DRN 5-HT neuronal activity remained intact while this residual response was reversed by MDL100907, a potent and selective 5-HT_2A_-Rs antagonist. Together, these findings emphasize the fact that the pharmacologic inactivation of the 5-HT_1A_ autoreceptor is necessary but likely not sufficient to fully prevent the acute inhibitory effects of SSRI on DRN 5-HT neuronal activity. The concomitant blockade of the 5-HT_1A_ and 5-HT_2A_-Rs is therefore required to prevent the undesired negative effects of SSRI on the serotonergic system (**Figure 2**).

There are alternative mechanisms by which the activation of the 5-HT_2A_-Rs might reduce the firing rate of DRN 5-HT neuronal activity. For example, it has been proposed that such an inhibitory action may also result from the activation of the 5-HT_2A_-Rs located on GABA interneurons in the LC (Szabo and Blier, 2001, 2002). In keeping with these data, evidence also suggested that the sustained administration of SSRI produced similar electrophysiological effects while antipsychotics displaying 5-HT_2A_-R antagonistic activity such as risperidone, reversed this attenuation in noradrenergic neuronal activity (Pandey et al., 2002; Dremencov et al., 2007). In light of the prominent excitatory NE innervation of the DRN (Baraban and Aghajanian, 1980; Vandermaelen and Aghajanian, 1983; Mongeau et al., 1997), the impairment of DRN 5-HT neuronal activity induced by DOI could be secondary to its inhibitory effect on LC NE neurons. In support of this latter hypothesis, we recently demonstrated in mice that the lesion of noradrenergic neurons with the neurotoxin DSP4 significantly attenuated DOI-induced decrease in DRN 5-HT neuronal activity (Quesseveur et al., 2013). Finally, it is important to note that 5-HT_2A_-Rs located in the PFC may also play a prominent role in the regulation of the DRN notably given the reciprocal anatomical and functional interactions between both regions. However, as mentioned above, evidence suggested that activation of cortical 5-HT_2A_-Rs increased the firing rate of DRN 5-HT neurons (Martin-Ruiz et al., 2001; Bortolozzi et al., 2003). To reconcile these findings with the fact that the systemic administration of DOI decreased 5-HT neuronal activity, it has been proposed that the 5-HT_2A_-R agonist would activate cortical pyramidal neurons projecting on GABAergic interneurons in the DRN (Serrats et al., 2005). Insomuch as the activation of 5-HT_2A_-Rs modulates the firing rate of DRN 5-HT, such activation could also result in changes in 5-HT release at the nerve terminals. In agreement with the fact that activation of the 5-HT_2A_-Rs reduces the firing activity of DRN 5-HT neurons, it has been demonstrated that the systemic administration of DOI to chloral hydrate-anesthetized rats reduced the extracellular 5-HT concentrations in the mPFC, an effect antagonized by MDL100907 (Martin-Ruiz et al., 2001).

It should be also noted that 5-HT_2A_-Rs might also participate in the regulation of the dopaminergic system through either direct or indirect mechanisms. In the VTA, 5-HT_2A_-Rs have also been identified in GABAergic interneurons, and their activation lead to the inhibition of dopaminergic activity (Doherty and Pickel, 2000; Nocjar et al., 2002). On the other hand, 5-HT_2A_-Rs might also be expressed directly onto DA VTA neurons and their activation would stimulate dopaminergic activity (Bubar et al., 2011; Howell and Cunningham, 2015). Hence, it has been shown that the systemic administration or local application of DOI increased the firing rate and burst firing of DA neurons as well as DA release in both the VTA and mPFC (Bortolozzi et al., 2005).

These electrophysiological and neurochemical data provide, at least in part, explanations of the fact that AAPs with 5-HT_2A_-R antagonistic activity, display antidepressant properties and are effective adjuncts in depressed patients responding inadequately to SSRIs (Blier and Szabo, 2005; Blier and Blondeau, 2011). There is indeed compelling clinical evidence for antidepressant efficacy of AAPs (Ghaemi and Katzow, 1999; Ostroff and Nelson, 1999; Hirose and Ashby, 2002; Shelton et al., 2005; Thase et al., 2007) and in the last few years, aripiprazole, olanzapine, and quetiapine have obtained FDA approvals for treatment of resistant depression in combination with SSRIs (DeBattista and Hawkins, 2009). Accordingly, it might be hypothesized that the progressive therapeutic activity of chronic treatment with SSRIs would be accompanied by a downregulation of 5-HT_2A_-Rs (Meyer et al., 2001). However, this assumption is still cause for debate (Massou et al., 1997; Zanardi et al., 2001; Muguruza et al., 2014).

## The 5-HT_2A_-Rs in the Regulation of Mood Related Behaviors and Antidepressant Response

### Preclinical Studies

A multitude of studies have associated 5-HT_2A_-Rs activation with depressive-like phenotypes. In behavioral paradigms relevant to depression, DOI significantly increased immobility time in the mouse FST, and this effect was abolished by a pre-treatment with MDL100907 (Diaz and Maroteaux, 2011). These results raised the possibility that 5-HT_2A_-R antagonists might produce antidepressant-like activities. Consistent with this hypothesis, it was shown that antisense-mediated downregulation of the 5-HT_2A_-Rs decreased the immobility of mice in the FST (Sibille et al., 1997) or that the 5-HT_2A_-R antagonists EMD281014 or MDL100907 produced similar antidepressant-like effects in rats (Zaniewska et al., 2010). More recently, a novel 5-HT_2A_-R antagonist BIP-1 has been synthesized and its acute or sustained administration was also shown to produce antidepressant-like activities not only in basal conditions but also in bulbectomized rats (Pandey et al., 2010) suggesting that the inactivation of 5-HT_2A_-Rs may also produce beneficial effects in animal models of depression. In order to confirm these results, we recently investigated whether the genetic ablation of 5-HT_2A_-Rs (5-HT_2A_^-/-^ mice) prevented chronic CORT-induced stress-related behavioral anomalies. Unexpectedly, the time of immobility in the TST was higher in 5-HT_2A_^-/-^ than in 5-HT_2A_^+/+^ wild-type (WT) in response to CORT administration (Petit et al., 2014). These results can therefore be interpreted as an exaggerated despair in 5-HT_2A_^-/-^ exposed to CORT. In this study, we did not find any basal modifications of despair in 5-HT_2A_^-/-^ mice as previously reported (Weisstaub et al., 2006) but our results suggested that the genetic inactivation of the 5-HT_2A_-R subtype is an important process to potentiate the depressive-like effects of chronic CORT administration. In agreement with this hypothesis, preclinical studies reported that chronic treatment with CORT desensitized the 5-HT_2A_-Rs within the paraventricular nucleus of the hypothalamus (Lee et al., 2009), whereas repeated stress decreased their density in the hippocampus (Schiller et al., 2003; Dwivedi et al., 2005). The mechanism by which glucocorticoids might have a repressive role on the 5-HT_2A_-R subtype is presently unclear, but recent investigations propose that glucocorticoids receptors may act directly as transcription factors at critical site of the *HTR2A* gene promoter (Falkenberg et al., 2011). Further studies exploring the reciprocal relationships between the HPA and the 5-HT_2A_-Rs are clearly required to provide a better understanding of how their interactions relates to the development of depression.

### Clinical Studies

Genetic association studies have focused on the genetic variants at the gene encoding for the 5-HT_2A_-Rs (Anguelova et al., 2003; Serretti et al., 2007). The association between MD and three single nucleotide polymorphisms (SNPs), G-to-A substitution at nucleotide -1438 (rs6311, -*1438G/A*), C-to-T substitution at nucleotide 102 (rs6313, *102C/T*) and C-to-T substitution at nucleotide 1354 (rs6314, His452Tyr, *1354C/T*) has been investigated, showing inconsistent results for the *C* allele of rs6313 (association: Zhang et al., 1997; Du et al., 2000; Arias et al., 2001a,b no association: Tsai et al., 1999; Minov et al., 2001; Zhang et al., 2008; Illi et al., 2009; Kishi et al., 2009; Wang et al., 2009), for the *A* allele of rs6311 (association: Enoch et al., 1999; Jansson et al., 2003; Lee et al., 2006; Christiansen et al., 2007; Kamata et al., 2011, opposite association: Choi et al., 2004, no association: Ohara et al., 1998; Illi et al., 2009; Kishi et al., 2009; Tencomnao et al., 2010), and for rs6314 which has been poorly studied (no association: Minov et al., 2001). Moreover, the functional consequences of these SNPs on 5-HT_2A_-R function and/or *HTR2A* expression remain poorly studied (Serretti et al., 2007), especially the *C* allele of rs6313, which could be submitted to methylation, a process known to prevent gene expression (Polesskaya et al., 2006) and for the *T* allele of rs6314 which could be associated with a decreased 5-HT_2A_-R-mediated intracellular signaling (Ozaki et al., 1997). We recently reported genetic arguments supporting an association between specific *HTR2A* SNPs and both susceptibility and severity of major depressive episodes in MD. Indeed, depressed patients with allelic variants suspected to decrease the expression/function of the 5-HT_2A_-Rs, i.e., the *C* allele of rs6313 and the rare *TT* variant of rs6314, have an increased severity of major depressive episodes (Petit et al., 2014). In this sample of depressed patients, the over-representation of rs6313 *C* carriers suggests that this allele was associated with MD. Moreover, a higher severity of major depressive episodes observed in *CT*/*CC* patients as compared to *TT* patients further supports the association of 5-HT_2A_-Rs and MD. Interestingly, in this sample of depressed patients, two patients carrying the rare *TT* genotype (452Tyr/Tyr) of rs6314 had severe melancholic major depressive episodes, but such association has not been reproduced in a recent study (Gadow et al., 2014). This might be related to the fact that the *TT* genotype has reduced ability to activate G proteins, downstream of 5-HT_2A_-Rs (Hazelwood and Sanders-Bush, 2004). Interestingly, the association of 5-HT_2A_-Rs and MD has been mainly reported in severe forms of suicide, notably such with suicidal attempts (Du et al., 2000; Giegling et al., 2006; Li et al., 2006; Saiz et al., 2008; Vaquero-Lorenzo et al., 2008) or melancholic features (Akin et al., 2004). The latter clinical results are also in line with those showing a greater 5-HT_2A_R binding in post-mortem brain tissue (Yates et al., 1990; Hrdina et al., 1993; Arranz et al., 1994; Pandey et al., 2002; Shelton et al., 2009) or in platelets (Hrdina et al., 1995, 1997; Sheline et al., 1995) from individuals with MD, and those evidencing that 5-HT_2A_-Rs mediated phosphoinositide synthesis was reduced in fibroblasts from patients with melancholic depression compared to controls (Akin et al., 2004).

It is noteworthy that variations in the gene encoding for the 5-HT_2A_-R have also been associated with the treatment outcome of SSRIs in MD (Choi et al., 2005; McMahon et al., 2006; Kato et al., 2009; Peters et al., 2009; Wilkie et al., 2009; Kishi et al., 2010; Lucae et al., 2010; Viikki et al., 2011). In particular, a recent pharmacogenetic study also pointed out that specific SNPs related with 5-HT_2A_-R signaling pathways might influence the therapeutic activity of SSRIs in Chinese patients with MD (Li et al., 2012). Unfortunately, in most cases the consequences of these polymorphisms on 5-HT_2A_-R expression and/or function are lacking knowledge and evidence.

## The 5-HT_2A_-Rs in the Regulation of Epilepsy and Antiepileptic Response

As we have highlighted in the previous paragraphs, 5-HT is an important neurotransmitter in the brain as it is involved in many neurological and psychiatric diseases including epilepsy. Serotonin receptors may directly or indirectly depolarize or hyperpolarize neurons by changing the ionic conductance and/or concentration within the cells (Barnes and Sharp, 1999). It is thus not surprising that 5-HT is able to change the excitability in most networks involved in epilepsy (Bagdy et al., 2007; Jakus and Bagdy, 2011; Gharedaghi et al., 2014).

Conventionally, epilepsy syndromes are classified into two distinct categories, focal and generalized, according to the seizure onset (arising from a specific brain area or from both hemispheres), the electroencephalogram and behavioral characteristics and the brain circuitry sustaining the paroxysms (Berg et al., 2010). Focal and generalized epilepsy differ also in the pathological neurochemical imbalance observed in the brain areas with a decrease and an increase of GABA function, respectively (Cope et al., 2009). This lead to a different therapeutic approach, indeed drugs that increase GABA concentration are first choice in focal/convulsive epilepsy and exacerbate absence epilepsy seizures. For instance, gabapentin, a structural GABA analog which increases GABA synthesis, is not indicated in generalized epilepsy syndromes (especially absence epilepsies), which it may exacerbate (Manning et al., 2003).

The majority of the focal and generalized seizures are convulsive (60%) while the remaining seizures are generalized non-convulsive. Moreover, since an obvious cell death or other tissue pathology is often absent, these epilepsies are idiopathic and typically associated with genetic abnormalities, an example of which is ASs (Crunelli and Leresche, 2002).

Here, we will focus on the focal TLE and the idiopathic generalized absence epilepsy. TLE is traditionally associated to many disorders localized to the cortex (neocortex and entorhinal cortex) and the hippocampal formation or both. Moreover, histological reports of TLE patients and animal models of epilepsy have consistently demonstrated that pathology is not limited to these areas but also to the thalamus, therefore the epileptogenic network in TLE is broad (Bernhardt et al., 2013). Typical ASs of idiopathic generalized epilepsies consist in sudden, brief periods of loss of consciousness which are accompanied by synchronous, generalized SWDs in the EEG (Crunelli and Leresche, 2002). SWDs originate from abnormal firing in thalamic and cortical networks and GABA_A_ inhibition is integral to their appearance (Crunelli and Leresche, 2002; Cope et al., 2009).

The involvement of the serotonergic system in epilepsy was suggested in the late 1950s (Bonnycastle et al., 1957) and all the areas involved in epilespy receive 5-HT innervetion and express different 5-HT-Rs including 5-HT_2A_-Rs (**Figure 1A**). Furthermore, 5-HT is known to regulate a wide variety of focal and generalized seizures, including absence epilepsy both in human and in animal models (Favale et al., 2003; Bagdy et al., 2007; Lorincz et al., 2007; Jakus and Bagdy, 2011). In general, agents that elevate extracellular 5-HT levels, such as 5-hydroxytryptophan and 5-HT reuptake blockers, inhibit both focal (limbic) and generalized seizures (Prendiville and Gale, 1993; Yan et al., 1994). Conversely, depletion of brain 5-HT lowers the threshold to audiogenically, chemically, and electrically evoked convulsions (Statnick et al., 1996). More recently, increased threshold to kainic acid-induced seizures was observed in mice with genetically increased 5-HT levels (Tripathi et al., 2008). These findings are corroborated by data showing that mice lacking the 5-HT_1A_- (Sarnyai et al., 2000; Parsons et al., 2001), 5-HT_2C_- (Applegate and Tecott, 1998), 5-HT_4_- (Compan et al., 2004) and, 5-HT_7_-Rs (Witkin et al., 2007), but also rats knocked-down for the 5-HT_2A_-Rs by antisense oligonucleotide treatment (Van Oekelen et al., 2003) are extremely susceptible to chemical and electrical-induced seizures. Nevertheless, since only 5-HT_2C_-R KO mice are prone to spontaneous death from seizures (Tecott et al., 1995), and seizures have not been reported with pharmacological blockade of different 5-HT-Rs, adaptive changes involving different mechanisms may play a role in the low seizure thresholds observed in 5-HT-R KO mice. In general, therefore, it seems that serotonergic neurotransmission by activating different 5-HT-Rs suppresses neuronal network hyperexcitability and seizure activity (Bagdy et al., 2007), although opposite effects have also been reported, especially for 5-HT_3-4-6-7_-Rs (Gharedaghi et al., 2014).

The role of pharmacological activation of 5-HT_2A_-Rs in epilepsy modulation is far from being well-established, however, it might be an important potential target in light of the recent evidence that their activation might be not only be anticonvulsant but also capable of reducing seizure-related mortality due to SUDEP (Buchanan et al., 2014), the leading cause of death in patients with refractory epilepsy (Shorvon and Tomson, 2011). In addition, we have recently shown that mCPP and lorcaserin, two preferential 5-HT_2C_-R agonists with different pharmacological profiles (Fletcher and Higgins, 2011; Higgins et al., 2013), stop the elongation of MDA and AD induced by repetitive perforant path stimulation recorded at the level of the granular cells of the hippocampal DG acting in urethane-anesthetized rats, an effect that was not blocked by SB242084, a selective 5-HT_2C_-R antagonist (Orban et al., 2014). The elongation of the MDA has been considered an electroencephalographic representation of epileptogenic phenomena occurring after the first electric insult (Stringer et al., 1989; Orban et al., 2013). Interestingly, preliminary results from our laboratory seem to indicate that mCPP and lorcaserin effects on MDA elongation might be due to the activation of 5-HT_2A_-rather than 5-HT_2C_-Rs since they were blocked by 5-HT_2A_-R antagonists while the 5-HT_2A_-R agonist TCB-2 mimicked mCPP and lorcaserin effects (unpublished observations). Conversely, evidence from other groups showed that DOI strongly facilitated kindling development and reduced the number of stimulations needed to produce generalized seizures in the amygdaloid kindled rats (Wada et al., 1997) while it was ineffective in any parameters on hippocampal partial seizures generated by low-frequency electrical stimulation of the hippocampus in rats (Watanabe et al., 1998). Similarly, Wada et al. (1992) showed that in the feline hippocampal kindled seizures, DOI had no effect displaying only a tendency to be anti-epileptic, decreasing the duration of AD and generalized tonic–clonic convulsions, although not significantly. In the same model, the selective 5-HT_2A_-R antagonist MDL100907, had no effect on seizure thresholds, secondary AD duration or latency of secondary AD (Watanabe et al., 2000). However, the 1 mg/kg dose of MDL100907 significantly increased the primary AD duration, suggesting that at this dose MDL100907 increased seizure severity in this model, although high AD control levels might have invalidated the 5-HT_2A_-R antagonist effect (Watanabe et al., 2000). The 5-HT_2A/2C_-R antagonist ketanserin and the more selective 5-HT_2A_-R antagonist ritanserin decrease the threshold for seizures maximal electroshock threshold (MEST) test in mice (Przegaliński et al., 1994). In other experimental models, 5-HT_2A_-R antagonists have failed to be effective in seizure control. Ritanserin was ineffective on kainic acid-induced seizures (Velisek et al., 1994) and ketanserin did not affect the seizure threshold for picrotoxin in mice (Pericic et al., 2005) or on ethanol withdrawal seizures (Grant et al., 1994), but antagonized cocaine-induced convulsions in a dose-dependent manner (Ritz and George, 1997). The 5-HT_2A/2C_-R and calcium antagonist dotarizine inhibited electroconvulsive shock (ECS)-induced seizures but had no effect on pentylenetetrazole (PTZ)-induced convulsions in rats (Lazarova et al., 1995) (**Table 1**).

**Table 1:** Role of the 5-HT2A receptors in temporal lobe epilepsy.

		Model	Effect	Reference
**Antiepileptic role of the 5-HT2A receptors in temporal lobe epilepsy**
Antagonists	MDL 11,939 (5-HT_2A_)	MEST test in *Lmx1b^*f/f/p*^* mice	Blocked DOI-TCB-2 effect in preventing seizure-induced respiratory arrest and death	Buchanan et al. (2014)
	Ketanserin (5-HT_2A_)	MEST test in mice	Decreases the threshold for seizures	Przegaliński et al. (1994)
	Ritanserin (5-HT_2A/2B/2C_)	MEST test in mice	Decreases the threshold for seizures	
	MDL 100907 (5-HT_2A_)	Electroshock-induced hippocampal partial seizures in rats	Increases primary AD duration	Watanabe et al. (2000)
Agonists	DOI (5-HT_2A/2C_) TCB-2 (5-HT_2A_)	MEST test in *Lmx1b^*f/f/p*^* mice	Prevented seizure-induced respiratory arrest and death	Buchanan et al. (2014)
	mCPP (5-HT_2A/2B/2C_)	MDA in rats	Stop MDA elongation (not blocked by SB242084)	Orban et al. (2014)
	Lorcaserin (5-HT_2B/2C_)	MDA in rats	Stop MDA elongation (not blocked by SB242084)	
	DOI (5-HT_2A/2C_)	Hippocampal kindled seizures in rats	Reduces AD duration	Wada et al. (1992)
**Pro-epileptic role of the 5-HT2A receptors**
Antagonists	Antisense oligonucleotide designed to inhibit 5-HT_2A_ expression	Tryptamine-induced serotonergic syndrome-associated convulsions	Inhibited tryptamine-induced bilateral convulsions and body tremors	Van Oekelen et al. (2003)
	MDL100907 (5-HT_2A_)	Feline hippocampal kindled seizures	No effect on seizure thresholds, secondary AD duration, or latency of secondary AD	Watanabe et al. (2000)
	Ritanserin (5-HT_2A/2B/2C_)	Kainic acid-induced seizures in rats	Has no effect	Velisek et al. (1994)
	Ketanserin (5-HT_2A_)	Cocaine-induced convulsions in mice	Dose-dependently inhibits seizures	Ritz and George (1997)
		Hippocampal kindled seizures in cats	Increases latency to generalized convulsions	Wada et al. (1992)
		Amygdala kindling in rats	Delays the development of kindling	Wada et al. (1997)
		Picrotoxin-induced seizures in stressed and unstressed mice	Has no effect on seizure thresholds	Pericic et al. (2005)
		Ethanol-withdrawal seizures in mice	Has no effect on seizure severity	Grant et al. (1994)
	Cinanserin (5-HT_2A/2C_)	Cocaine-induced convulsions in mice	Dose-dependently inhibits seizures	Ritz and George (1997)
	Pirenperone (5-HT_2A/2C_)	Cocaine-induced convulsions in mice	Dose-dependently inhibits seizures	
	Dotarizine (5-HT_2A/2C_)	Electroshock-induced seizures in rats	Increases the threshold for seizures	Lazarova et al. (1995)
		PTZ-induced seizures in rats	Has no effect on seizure thresholds	
Agonists	DOI (5-HT_2A/2C_)	Hippocampal kindled seizures in cats	Decreases latency to generalized convulsions	Wada et al. (1992)
		Amygdala kindling in rats	Facilitates kindling and reduces the number of stimulations needed to elicit generalized convulsions	Wada et al. (1997)
		Picrotoxin-induced seizures in stressed and unstressed mice	Has no effect on seizure thresholds	Pericic et al. (2005)

*MEST, maximal electroshock threshold; PTZ, pentylenetetrazole; SE, status epilepticus.*

As far as the 5-HT control of generalized ASs is concerned, most of the limited available evidence has been obtained in WAG/Rij rats, with 5-HT_1A_-, 5-HT_2C_-, and 5-HT_7_-Rs appearing as the most critical for the expression of this form of epilepsy (Bagdy et al., 2007). Briefly, activation or inhibition of 5-HT_1A_- and 5-HT_7_-Rs increases or decreases ASs, respectively, while 5-HT_2C_-R agonists are effective in inhibiting epileptiform activity and 5-HT_2C_-R antagonism lacks any effects (Jakus et al., 2003; Jakus and Bagdy, 2011). In agreement with this evidence, fluoxetine, and citalopram caused a moderate increase in SWDs; potentiated or inhibited by pre-treatment with SB-242084 and the 5-HT_1A_-R antagonist WAY-100635, respectively (Jakus and Bagdy, 2011). The role of 5-HT_2A_-Rs has not instead been investigated in WAG/Rij rats yet. In another genetic animal model of absence epilepsy, the groggy (GRY) rats, increasing 5-HT levels by treatment with the 5-HT reuptake inhibitors fluoxetine and clomipramine, inhibits SWD generation, an effect mimicked by DOI and blocked by ritanserin pre-treatment (Ohno et al., 2010). Consistently, in atypical ASs induced by AY-9944, DOI reduced the total duration and number of SWDs, and ketanserin exacerbated the number of SWDs. On the other hand, in contrast to the evidence obtained in WAG/Rij rats, 5-HT_2C_-R activation by mCPP had no effect on total duration or number of SWD in this model of atypical absence epilepsy (Bercovici et al., 2007).

In contrast to these findings, however, earlier evidence had shown that serotonergic neurotransmission and 5-HT_2A_-Rs do not appear to be involved in the pathogenesis or control of ASs in the most widely used rat model of absence epilepsy, the GAERS (Danober et al., 1998) (**Table 2**). Although this discrepancy could be simply due to differences between the two experimental models, it is more likely explained by the lack of selectively of the serotoninergic drugs that were used in the earlier study in GAERS. The role of 5-HT, and especially the different areas in which the modulation of ASs might occur, has not been examined thoroughly and it is currently object of investigation in our laboratories. Since we have recently shown that an aberrant eGABA function in VB neurons is a necessary factor in the expression of SWDs associated with typical absence epilepsy (Cope et al., 2009; Di Giovanni et al., 2011; Errington et al., 2011, 2014), it is conceivable that some of the systemically injected 5-HT ligand effects on ASs (Danober et al., 1998; Isaac, 2005; Bagdy et al., 2007; Bercovici et al., 2007; Ohno et al., 2010) occur via a modulation of tonic GABA_A_ inhibition. This hypothesis is based also on the evidence that DA and especially the activation of D_2_-Rs decreases both ASs (Deransart et al., 2000) and eGABA current in GAERS VB neurons (Yague et al., 2013; Crunelli and Di Giovanni, 2014). Indeed, our preliminary results show that 5-HT_2A_-R ligands lack any effect on phasic synaptic GABA_A_ inhibition in VB thalamocortical neurons of Wistar rats (Cavaccini et al., 2012), while 5-HT_2A_-R selective agonists significantly enhanced the tonic eGABA_A_ conductance. This enhancement of eGABA_A_ tonic current was blocked by co-application of 5-HT_2A_-R antagonists which were devoid of any effect *per se*. Strikingly, 5-HT_2A_-R antagonists were instead effective in decreasing the aberrant GABA_A_ tonic current in GAERS. From these findings, we can speculate that the activation of the 5-HT_2A_-Rs would have a pro-epileptic activity, although this evidence has not been obtained yet* in vivo*.

**Table 2:** Role of the 5-HT2A receptors in absence epilepsy.

			Model	Effect	Reference
Typical absence epilepsy	Agonists	DOI (5-HT_2A/2C_)	GRY rats	Inhibits SWDs	Ohno et al. (2010)
		m-CPP (5-HT_2A/2B/2C_)	WAG/Rij rats	Decreases the duration and frequency of SWDs	Jakus et al. (2003)
	Antagonists	Ritanserin (5-HT_2A/2B/2C_)	GRY	Increases SWDs	Ohno et al. (2010)
		Ritanserin (5-HT_2A/2B/2C_)	GAERS	Has no effect	Marescaux et al. (1992)
		Ketanserin (5-HT_2A_)	GAERS	Has no effect	
Atypical absence epilepsy	Agonists	DOI (5-HT_2A/2C_)	AY-9944 rats	Reduces the frequency and duration of slow SWDs	Bercovici et al. (2007)
	Antagonists	m-CPP (5-HT_2A/2B/2C_)	AY-9944 rats	Has no effect	Bercovici et al. (2007)
		Ketanserin (5-HT_2A_)	AY-9944 rats	Increases the frequency and duration of slow SWDs	Bercovici et al. (2007)

*GRY, groggy, WAG/Rij, Wistar Albino Glaxo rats from Rijswijk; SWD, spike-wave discharge; GAERS, Genetic Absence Epilepsy in Rats from Strasbourg; AY-9944, *trans*-*N*, *N*-bis[2-chlorophenylmethyl]-1,4-cyclohexanedimethanamine dihydrochloride. Modified from Gharedaghi et al. (2014).*

There is evidence indicating that 5-HT2A-R activation potentiates the inhibitory effect of lamotrigine, a widely used antiepileptic agent, on voltage-gated sodium channels (Than et al., 2007). Lamotrigine is the only other antiepileptic drug (AED) with clear benefit for bipolar disorder, and is approved by FDA for maintenance treatment (Bowden et al., 2003). Interestingly, a study in Long-Evans rats with spontaneous SWDs has indicated that chronic lamotrigine treatment can benefit patients with absence epilepsy via suppression of seizures and amelioration of comorbid anxiety and depression (Huang et al., 2012).

Further, some ligand-binding studies in animals have shown that the antiepileptic valproate increases 5-HT_2A_-R expression (Green et al., 1985; Sullivan et al., 2004), although an *in vivo* imaging study has not confirmed it in acute mania (Yatham et al., 2005). This study, however, cannot exclude the possibility that valproate improves mood symptoms by altering second messenger signaling cascades linked to 5-HT_2A_-Rs. Indeed, brain 5-HT_2A_-Rs are coupled via G-proteins to phosphoinositol pathway, and there is a growing body of evidence which suggests that both valproate and lithium have multiple effects on this pathway (Brown and Tracy, 2013).

The abovementioned studies show that generally 5-HT has an anticonvulsant effect in both generalized and focal epilepsy and the 5-HT2-Rs appear to play a major role, although contrasting evidence also exists. In particular, the anti- versus pro-epileptic effects of the 5-HT2A-Rs might depend on the dose of the ligands used, with pro-convulsive effects when the receptors are excessively activated, the experimental model investigated and different populations of receptors. Moreover, at high doses, the selectivity of these ligands is lost and other mechanisms cannot be ruled out.

More research is needed to clarify the role of 5-HT2A-Rs in seizures especially in absence epilepsy. Thus, increasing our understanding of the role of 5-HT2A-Rs and their modulation of other neurotransmitter systems such as GABA might reveal a new possible therapeutic mechanism with potential translational significance.

## Do the 5-HT2A-Rs Play a Role in the Comorbidity between Epilepsy and Depression?

It is estimated that between 15 and 30% of people with epilepsy develop several psychiatric disorders, such as anxiety, depression, and different levels of cognitive impairments (Stafford-Clark, 1954; Kanner and Balabanov, 2002; Kanner, 2003). The patients with partial complex epilepsy, such as TLE, or who have poorly controlled epilepsy have the highest frequency rate of comorbid affective disorders (Kanner et al., 2012). Besides, depression-like behavior has also been found in generalized epilepsy such as childhood absence epilepsy (Vega et al., 2011). This clear link between epilepsy, comorbid psychiatric disorders and monoaminergic and specifically serotoninergic dysfunction has been also observed in humans (Harden, 2002) and different animal models of epilepsy (Sarkisova and van Luijtelaar, 2012; Epps and Weinshenker, 2013). Moreover, the animal and human evidence has revealed that the relationship between depression and epilepsy is in reality bidirectional. Indeed patients with depression and especially suicide attempters have an increased seizure risk compared to the normal population (Hesdorffer et al., 2006). Thus, the fact that epilepsy and depression may share common pathogenic mechanisms and dysfunction of the serotonergic system is an obvious explanation for this bidirectional comorbidity, since defects in the serotonergic system are linked to both conditions (Epps et al., 2012; Epps and Weinshenker, 2013). In agreement, we have showed further evidence of the involvement of both serotonergic and dopaminergic systems in the pathogenesis of epilepsy (Cavaccini et al., 2012; Orban et al., 2013; Yague et al., 2013; Connelly et al., 2014; Crunelli and Di Giovanni, 2014; Orban et al., 2014), in depression and its pharmacological treatments (Di Giovanni, 2008; Esposito et al., 2008). Compelling evidence for the involvement of 5-HT_1A_- and 5-HT_7_-Rs in epilepsy and depression has been described, therefore it is possible to infer that agonists at these receptors might have both antiepileptic and antidepressant activity with also cognitive enhancer efficacy (Orban et al., 2013). On the other hand, the role of the other 5-HT_2A_-Rs has been less investigated, and this field is still in its infancy with many issues that still need to be addressed. Regarding the 5-HT_2A_-R as a drug target for treating depression and epilepsy, it has recently been shown in WAG/Rij rats that sub-chronic treatment with aripiprazole, a new antipsychotic with antagonism at 5-HT_2A_/5-HT_6_-Rs and also partial agonism at D_2_ DA and 5-HT_1A_ and 5-HT_7_-Rs, has an anti-AS effect, and positive modulatory actions on depression, anxiety, and memory which might also be beneficial in other epileptic syndromes (Russo et al., 2013). Nevertheless, this study did not identify which receptor subtype underlined these promising aripiprazole therapeutic properties. Perhaps, the 5-HT-Rs more directly linked with the antidepressant and antiepileptic effects of aripiprazole might be the 5-HT_1A/7_-Rs, in light of the well-known effects of clozapine on seizures. Clozapine, the first AAP to be developed with some 5-HT_2A_-R antagonist effects, increases seizure risk even at therapeutic serum levels (Hedges et al., 2003) and it is indeed the only psychotropic drug to have received a FDA black box warning regarding seizures.

Improved seizure control has also been observed in epileptic patients treated for psychiatric disorders with antidepressants elevated extracellular serotonin in the epileptic foci can lead to an anticonvulsant effect (Specchio et al., 2004), but the contribution of the single 5-HT-Rs has not yet been revealed.

As far as cognitive impairments are regarded, preclinical studies have shown that the 5-HT_2A_-R activation also has some therapeutic benefits. For instance, ketanserin inhibited the impairment of short-term memory which is seen after seizures studied by spontaneous alternation rat behavior in the Y-maze task (Hidaka et al., 2010). In addition, ketanserin inhibited ECS-induced retrograde amnesia in the step-down passive avoidance task, suggesting that 5-HT_2A_-Rs impede consolidation and/or retrieval of memory after seizures (Genkova-Papazova et al., 1994) (**Table 3**).

**Table 3:** 5-HT_2A_ receptors in comorbidity between epilepsy and depression.

	Model	Effect	Reference
Lamotrigine	Chronic pain states in rats	+ m-CPP (5-HT2A/2B/2C) increased the reflex inhibitory action of lamotrigine	Than et al. (2007)
Lamotrigine	Chronic pain states in rats	Decreased the reflex inhibitory action of + Ketanserin (5-HT_2A_) lamotrigine	
Lamotrigine	Humans	Bipolar disorders	Bowden et al. (2003)
Lamotrigine	WAG/Rij rats	Suppression of AS and amelioration of comorbid anxiety and depression	Huang et al. (2012)
Aripiprazole (5-HT2A/5-HT6 antagonist)	WAG/Rij rats	Suppression of AS amelioration of comorbid anxiety depression and memory impairment	Russo et al. (2013)
Valproate	Humans	Increases 5-HT2A-R expression	Green et al. (1985); Sullivan et al. (2004)
Valproate	ECS	Inhibited impairment of spontaneous alternation behavior	Hidaka et al. (2011)
SSRIs (5-HT-R?)	Different models	Anticonvulsant	Specchio et al. (2004)
Ketanserin (5-HT_2A_ antagonist)	ECS	Inhibited the impairment of short-term memory	Hidaka et al. (2010)
Ketanserin (5-HT_2A_ antagonist)	ECS	Inhibited electroconvulsive shock-induced retrograde amnesia	Genkova-Papazova et al. (1994)

*WAG/Rij, Wistar Albino Glaxo rats from Rijswijk; ECS, electroconvulsive shock.*

Summarizing, both agonists and antagonists appear to be useful in epilepsy treatment (**Tables 1** and **2**). These paradoxical actions of 5-HT_2A_ antagonists and agonists can be reconciled taking in to consideration that both agonism and antagonism induce 5-HT2A-Rs desensitization or downregulation (Gray and Roth, 2001). The main hindrance for the development of 5-HT_2A_-R agonists is the hallucinogenic effects (Krebs-Thomson et al., 1998). New 5-HT_2A_ compounds with higher selectivity and which lack these aversive side effects are needed.

## Conclusion

Together, the observations reviewed here support an important role for 5-HT_2A_-Rs in both affective disorders and normal and pathologic neuronal excitability. The available literature suggests that the antagonism at 5-HT_2A_R might have beneficial effects on both disorders. Moreover, 5-HT_2A_-R antagonists might represent a new therapeutic strategy in epileptic patients with comorbid depression and cognitive dysfunctions. In addition, 5-HT_2A_-R antagonism may improve the effectiveness of medical therapy with respect to seizure control for both focal and generalized seizures if they are combined with existing AEDs and/or SSRIs. The pathophysiology of depression and epilepsy might result, at least in part, directly from a dysregulation of brain serotonin 2A neurotransmission or indirectly from the dysfunction of other neurotransmitter systems (i.e., dopaminergic, glutamatergic, GABAergic) that are under 5-HT_2A_ control. Needless to say, it remains to be determined whether epilepsy and its comorbid psychiatric disorders are instead mere epiphenomena of the primary alteration of 5-HT_2A_-R signaling.

## Conflict of Interest Statement

The authors declare that the research was conducted in the absence of any commercial or financial relationships that could be construed as a potential conflict of interest.

## Abbreviations

AAP: atypical antipsychotic
AD: after discharge
5-HT: 5-hydroxytryptamine or serotonin
5-HT_2A_-Rs: serotonin 2A receptors
ASs: absence seizures
BLA: basolateral amygdala
CORT: corticosterone
DA: dopamine
DG: dentate gyrus
DOI: 2,5-dimethoxy-4-iodoamphetamine
DRN: dorsal raphe nucleus
eGABA: extrasynaptic GABA_A_
FST: forced swim test
GAD: glutamic acid decarboxylase
GAERS: Genetic Absence Epilepsy in Rats from Strasbourg
GPCRs: G protein-coupled receptors
LC: locus coeruleus
MD: major depression
MDA: maximal dentate activation
mPFC: medial prefrontal cortex
MRN: medial raphe nucleus
NE: norepinephrine
PAG: periaqueductal gray
PFC: prefrontal cortex
SERT: serotonin transporter
SSRI: selective serotonin reuptake inhibitor
SUDEP: sudden unexpected death in epilepsy
SWDs: spike and wave discharges
TLE: temporal lobe epilepsy
TST: tail suspension test
VB: ventrobasal thalamus
VTA: ventral tegmental area
